# Smoking Prevalence Increases following Canterbury Earthquakes

**DOI:** 10.1155/2013/596957

**Published:** 2013-11-07

**Authors:** Nick Erskine, Vivien Daley, Sue Stevenson, Bronwen Rhodes, Lutz Beckert

**Affiliations:** ^1^Department of Medicine, University of Otago, Christchurch 8140, New Zealand; ^2^Canterbury District Health Board, Department of Respiratory Medicine, Christchurch 8041, New Zealand

## Abstract

*Background*. A magnitude 7.1 earthquake hit Canterbury in September 2010. This earthquake and associated aftershocks took the lives of 185 people and drastically changed residents' living, working, and social conditions. *Aim*. To explore the impact of the earthquakes on smoking status and levels of tobacco consumption in the residents of Christchurch. *Methods*. Semistructured interviews were carried out in two city malls and the central bus exchange 15 months after the first earthquake. A total of 1001 people were interviewed. *Results*. In August 2010, prior to any earthquake, 409 (41%) participants had never smoked, 273 (27%) were currently smoking, and 316 (32%) were ex-smokers. Since the September 2010 earthquake, 76 (24%) of the 316 ex-smokers had smoked at least one cigarette and 29 (38.2%) had smoked more than 100 cigarettes. Of the 273 participants who were current smokers in August 2010, 93 (34.1%) had increased consumption following the earthquake, 94 (34.4%) had not changed, and 86 (31.5%) had decreased their consumption. 53 (57%) of the 93 people whose consumption increased reported that the earthquake and subsequent lifestyle changes as a reason to increase smoking. *Conclusion*. 24% of ex-smokers resumed smoking following the earthquake, resulting in increased smoking prevalence. Tobacco consumption levels increased in around one-third of current smokers.

## 1. Background 

On Saturday, September 4, 2010, at 4:35 a.m., a magnitude 7.1 earthquake struck the Canterbury region of New Zealand. Nearly six months later on Tuesday 22 February 2011 at 12:51 pm, a second earthquake measuring magnitude 6.3 struck Christchurch, the major city in Canterbury. Although lower on the magnitude scale, the intensity, acceleration, and violence of the ground shaking were among the strongest ever recorded in an urban area, and 185 people lost their lives. The earthquake caused considerable destruction and significantly changed residents' living, working, and social conditions.

The smoking rate in New Zealand in 2009 was approximately 21.8% [1]. Smoke-free initiatives have been successful in New Zealand, with strengthened constraints on advertising and displaying tobacco products resulting in reduced prevalence rates and significant decreases in youth uptake of smoking [1]. Recently, in response to calls from the New Zealand Maori Party for a general smoking ban [2], the government approved a longer term goal to make New Zealand essentially a smoke-free nation by 2025 [3]. 

Despite these national successes, staff working at the Canterbury District Health Board hospitals noticed that more patients seemed to be smoking after the earthquakes. The aim of this study was to verify any change in smoking status of Canterbury residents and to explore changes in levels of tobacco consumption following the earthquakes. 

## 2. Methods

The study was conducted in Christchurch, the largest city in the South Island of New Zealand, with a population of approximately 380,000. Because of widespread displacement of thousands of residents, a randomised census based survey was not possible. Semistructured interviews were conducted with participants in high flow pedestrian areas, including two shopping malls and the new temporary bus exchange. The study was conducted over a two-month period in December 2011 and January 2012, approximately 15 months since the earthquake activity started in Canterbury with a 7.6 quake. Participants were asked to give up two to three minutes of their time to complete a questionnaire. All interviews were conducted by one author (Nick Erskine). 

This study was approved by the local ethics committee of the Ministry of Health with approval number URB/11/EXP/024.

## 3. Results

A total of 1,001 participants agreed to take part in the survey. We interviewed a total of 428 (42.7%) males and 573 (57.3%) females.

Most participants were NZ European, with Maori, Pacific Island, and Asian ethnicities represented in numbers which approximate proportions in the general population (Table 1).

### 3.1. Smoking Status

August 2010 was used as the anchor date as Christchurch had not experienced any major earthquakes prior to this. Participants were asked about their smoking status in August 2010 and then to comment on the changes in their smoking habits since (Figure 2).

The total number of people who had never smoked (defined as less than 100 cigarettes in their lifetime) was 409 (41%), 273 (27%) were current smokers, three people could not recall their smoking status, and 316 (32%) had smoked but were ex-smokers in August 2010; 76 (24%) of these ex-smokers had smoked at least one cigarette since the earthquakes started (Figure 3). Of these relapsed ex-smokers, 31 (41%) smoked less than 20 cigarettes in total following the first quake, 16 (21%) smoked between 21 and 100 cigarettes, and 29 (38%) smoked more than 100 cigarettes and had essentially resumed their old smoking habits.

### 3.2. Tobacco Consumption Levels

Of the 273 people who were smoking in August 2010, 93 (34.1%) reported increased consumption, 94 (34.4%) were smoking the same amount, and 84 (31.5%) reported decreased consumption (Figure 4). Of the 93 participants who increased their cigarette consumption since August 2010, 69 offered a reason for change, with 53 (76.8%) of these quoting the earthquakes and associated stress factors to be responsible for the increase in their smoking habit. Other reasons given were general desire to smoke more and influence of work related stress factors. 

Of the 84 participants who were smoking less following the earthquakes, 63 offered a reason for the change. Of these, six (9.5%) believe the earthquake contributed to their decrease in consumption, with four citing the lack of social opportunities available in Christchurch as their reason. The most common reasons for quitting were given as health concerns, pressure from family, and friends and price increases.

## 4. Discussion

The most important finding from this study was that around a quarter of ex-smokers resumed smoking following the earthquakes and that of the third of people who increased their tobacco consumption, over half cited the earthquakes as the reason for the increase.

### 4.1. The Sample

**Figure 1:**
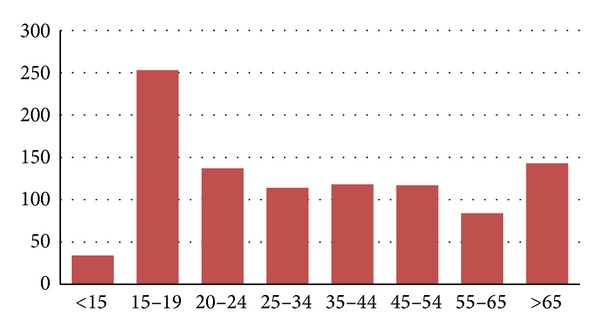
Age distribution in years of the 1001 participants who participated in the survey. Note the slight overrepresentation of the 15–19 year age group.

**Figure 2:**
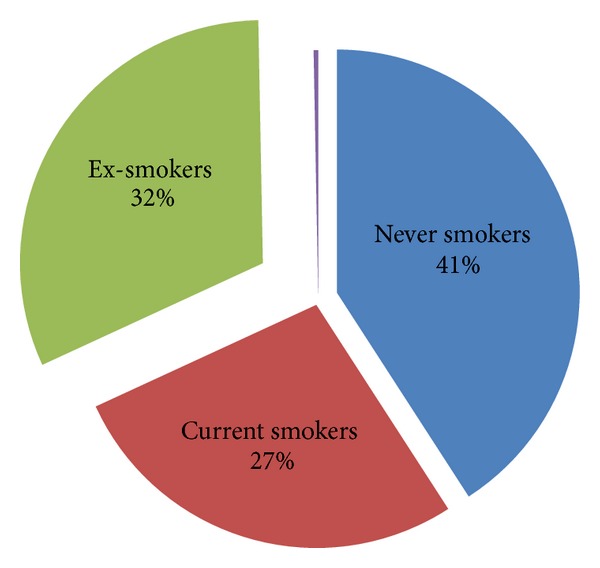
Prior to the Christchurch earthquake, 409 (41%) participants were never smokers, 316 (32%) were ex-smokers, and 273 (27%) were current smokers. Three participants (0.3%) were not sure about their smoking status in August 2010.

**Figure 3:**
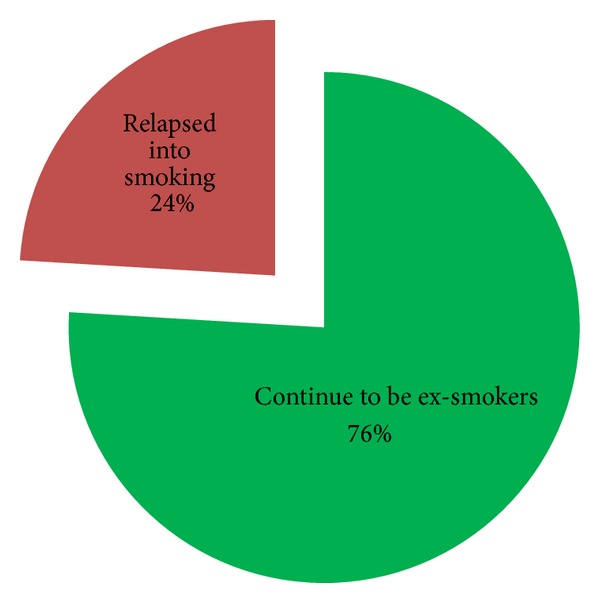
Of the 316 ex-smokers at the time of the earthquake 76 (24%) relapsed into their smoking habit following the earthquakes.

**Figure 4:**
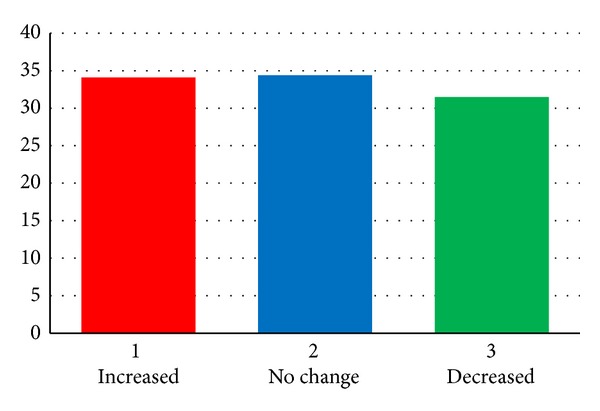
Change of smoking level in 2012 compared to the baselines smoking level in August 2010 in participants who continued to smoke.

Conducting rigorous research following this disaster was highly problematic. Randomised census-based research was impossible, with many thousands of residents displaced from their census addresses. The 2011 NZ census, planned for February 2011, was cancelled because Canterbury was in a state of civil emergency for several months, and government agencies were focusing on providing practical support and help. The central city was fenced off, businesses closed or relocated to the outskirts, and people worked from cars, cafes, homes, and even tents. The survey locations (two shopping malls and the temporary bus exchange) were chosen strategically to provide the most representative snapshot possible, but the cohort surveyed showed a bias in favour of young people (see Figure 1). Despite this bias, this data is probably the best snap shot of smoking prevalence that could be achieved after a natural disaster on this scale.

The 2006 census reported an average smoking rate of 17.4% for men and 20.2% for women in the Canterbury region which, at an overall rate of 18.8%, is slightly lower than the national average [4]. Our data shows that the smoking rate in August 2010 was 27%, which is higher than the national average. Some of this increased higher smoking rate may be explained by the overrepresentation of the 15–19 and 20–24 age groups, which have national smoking rates of 19% and 30%, respectively [4, 5]. This overrepresentation does not impact on the important finding that 24% ex-smokers resumed their smoking habit. 

### 4.2. Effective Public Health Strategies Persist

New Zealand has the goal of proactively reducing smoking prevalence and tobacco availability to minimal levels, thereby making New Zealand essentially a smoke-free nation by 2025 [3]. Public health strategies have been implemented with increasing success over many years. These include regular tobacco prices increases, organisations implementing smoke-free policies, creating smoke-free green spaces, and supporting people to quit by providing highly subsidised cessation medications, a national telephone quit line, and systematic cessation interventions for patients in primary and secondary care. Despite earthquakes these public health strategies continued to have an impact on Christchurch as participants most often cited health concerns, pressure from family and friends, and price increases as the reasons to reduce their cigarette consumption. These reasons were well aligned with the data found in the 2009 New Zealand Tobacco Use Survey, namely, concerns about their own health, cost, sick of smoking, and pressure from friends and family [1]; although in our survey, pressure from friends and family was rated higher than it was in the national data. This suggests that public health strategies to reduce cigarette consumption continue to be effective to some degree, even when an area is affected by a natural disaster.

### 4.3. Stress as a Mediating Factor

Virtually all patients who relapsed into their smoking habit quoted the earth quake and associated stress as a reason to take up smoking again. The stress coping model of substance use suggests that cigarettes help people cope throughout stressful situations by regulating mood [6]. This model is supported by other reports correlating periods of high stress and smoking levels [7], smokers' subjective reports that smoking helps them relieve tension [8], and the lower prevalence of other stress management behaviour in relapsed and recalcitrant smokers [9]. The extreme levels of stress created by the Canterbury earthquakes may explain the increased consumption despite public health interventions. The number of people who have relapsed in their smoking habit during this period is consistent with previous studies which examined stress as a trigger for lapsing [8] and laboratory studies demonstrating the effect of stress in decreasing the ability to resist smoking [10]. Although there are many studies examining the impact of stress on smoking behaviour, there are few studies reporting on natural disasters influencing smoking habits. 

### 4.4. Other Disasters and Smoking

Following Hurricane Katrina, Flory et al. found higher than predicted smoking rates in Hurricane Katrina survivors [11]. Jiao et al. examined characteristics of myocardial infarction patients, comparing pre- and post-Hurricane Katrina populations and found that the postevent population had a much higher smoking prevalence [12]. However, neither study followed an individual's change in smoking status over the time of the disaster. Reviewing the international literature we are not aware of any baseline relapse rate of long term ex-smokers. An Australian study managed to capture this data in a group of young adults where most were affected by the Canberra bushfires in 2003 [13]. A longitudinal epidemiological study had recorded smoking status in 1999-2000 and follow-up data 4 years later showed that 13.2% had started, continued, or increased their smoking. They found a positive correlation between the degree of trauma experienced and an increase in tobacco consumption. Our study design allowed us to report on increased tobacco consumption and new onset of smoking separately. We found that the increase in tobacco consumption in some participants was balanced by reduced tobacco consumption in others. The rate of uptake of smoking is higher than the rate reported in the Australian study. This may be an effect of the timing of the interview as aftershocks were still happening during our interview period, while the Australian “second wave” was well after the event. 

## 5. Conclusion

This study found that 24% of people who were not smoking prior to the earthquakes resumed their smoking habit following this natural disaster. One-third of people currently smoking at the time of the first earthquake increased their tobacco consumption, with earthquake and aftershocks most often quoted as the reason for the increased consumptions. One-third of current smokers decreased their tobacco consumption, with concern for own health, cost, and peer pressure as the most quoted reasons. These findings will inform natural disaster planning and the need to provide additional cessation supports in the aftermath of a disaster. It also indicates a need for cessation services to arm their clients with alternative ways to relieve stress. Public health services should continue to provide consistent smoke-free information, as some people (possibly those less affected personally) are still able to receive this and act on it.

## Figures and Tables

**Table 1:** The ethnicity question replicated the question used in the New Zealand census: “which ethnic group or groups do you belong to?” Some participants associated with more than one ethnic group.

Ethnic distribution of participants	
New Zealand European	774
Mäori	85
Samoan	25
Cook Island Mäori	2
Tongan	3
Niuean	1
Chinese	18
Indian	13
Other (e.g., Dutch, Japanese, and Tokelauan)	98
Do not know/unsure	0
Refused	6
